# Differences in pulmonary flow patterns between surgical and percutaneous implanted bovine valves to restore the right ventricle outflow tract continuity: a four dimensional flow magnetic resonance study

**DOI:** 10.1186/1532-429X-16-S1-O44

**Published:** 2014-01-16

**Authors:** Soha Romeih, Rob J van der Geest, Arno Roest, Anje M Spijkerboer, Barbara J Mulder, Nico A Blom, Maarten Groenink

**Affiliations:** 1 Radiology, AMC, Amsterdam, Netherlands; 2 Radiology, Leiden University Medical Center, Leiden, Netherlands; 3 Cardiology, Academic Medical Center, Amsterdam, Netherlands; 4 Paediatrics, Leiden University Medical Center, Leiden, Netherlands

## Background

In patients with congenital heart defects, restoration of the right ventricle outflow tract (RVOT) continuity by surgical or percutaneous bovine valve implantation is a common procedure. Flow characteristics in such valves are complex, therefore the aim of this study was demonstrating the potential application of 4D MRI flow for the comprehensive assessment of pulmonary flow patterns after surgical and percutaneous bovine valve implantation in the RVOT.

## Methods

Fifteen patients after percutaneous bovine valve implantation (17.2 ± 2.0 years), 15 patients after surgical implantation (15.8 ± 1.7 years), and 15 healthy volunteers (as a control group) (16.5 ± 1.5 years) were included. All subjects underwent a comprehensive cardiac MRI protocol. Analysis focused on the presence of vertical flow patterns; pulmonary flow eccentricity and wall shear rate (WSR) assessment.

## Results

**Figure 1:**
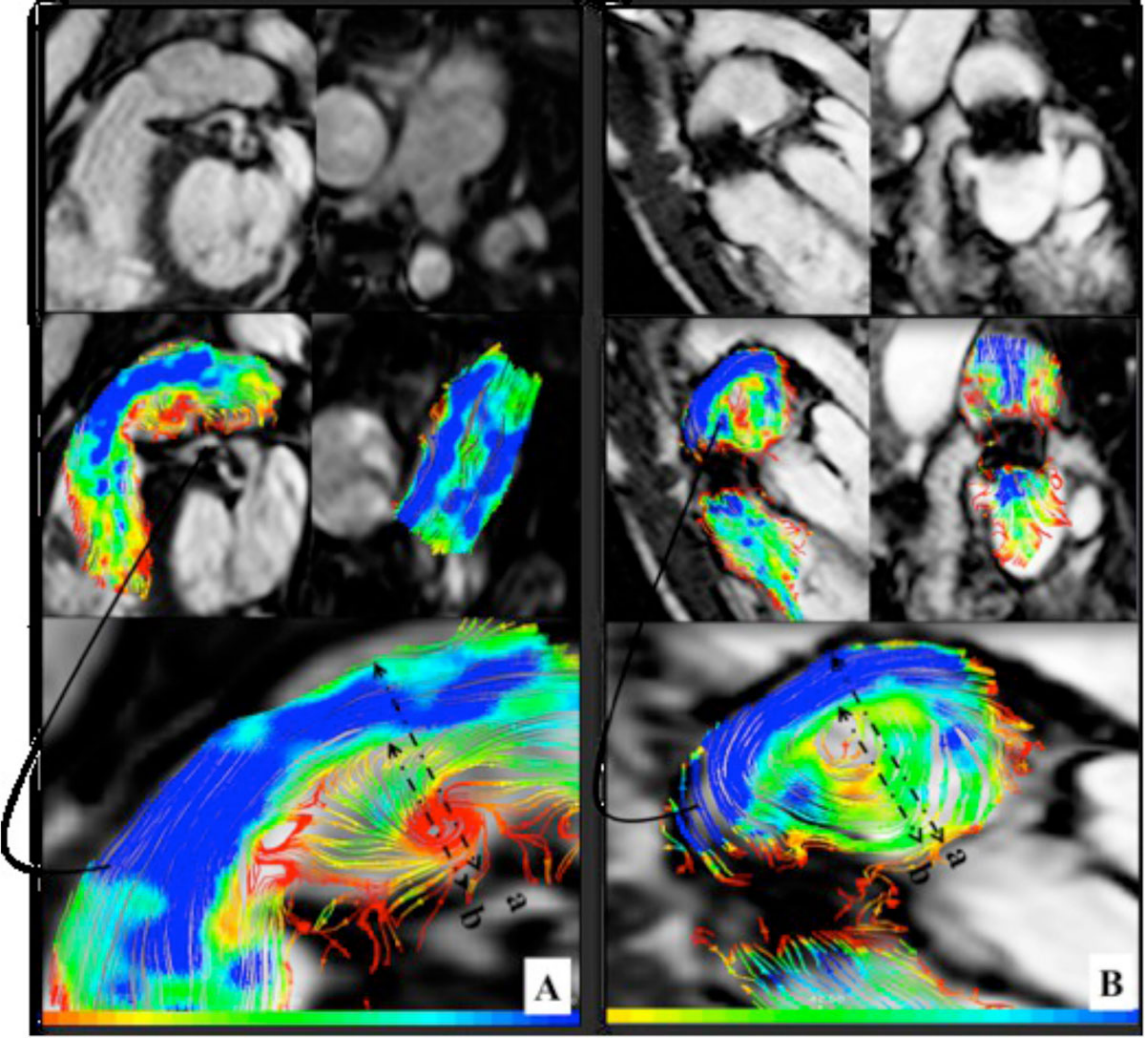
**Upper rows; (A) Contegra conduit, upper left (sagittal view), upper right (coronal view); there is a focal small aneurysm in the lateral wall**. (B) Melody valve, upper left (sagittal view), upper right (coronal view). Middle rows show traces of color-coded streamlines demonstrating eccentric flow, pulmonary flow is deviated from the midline, and vortex is seen. The lower rows are magnification of the vortex in a phase with the maximal vortex diameter. Vortex size measured as a percentage of the vessel diameter: diameter of the vortex (b)/diameter of pulmonary artery (a).

**Figure 2:**
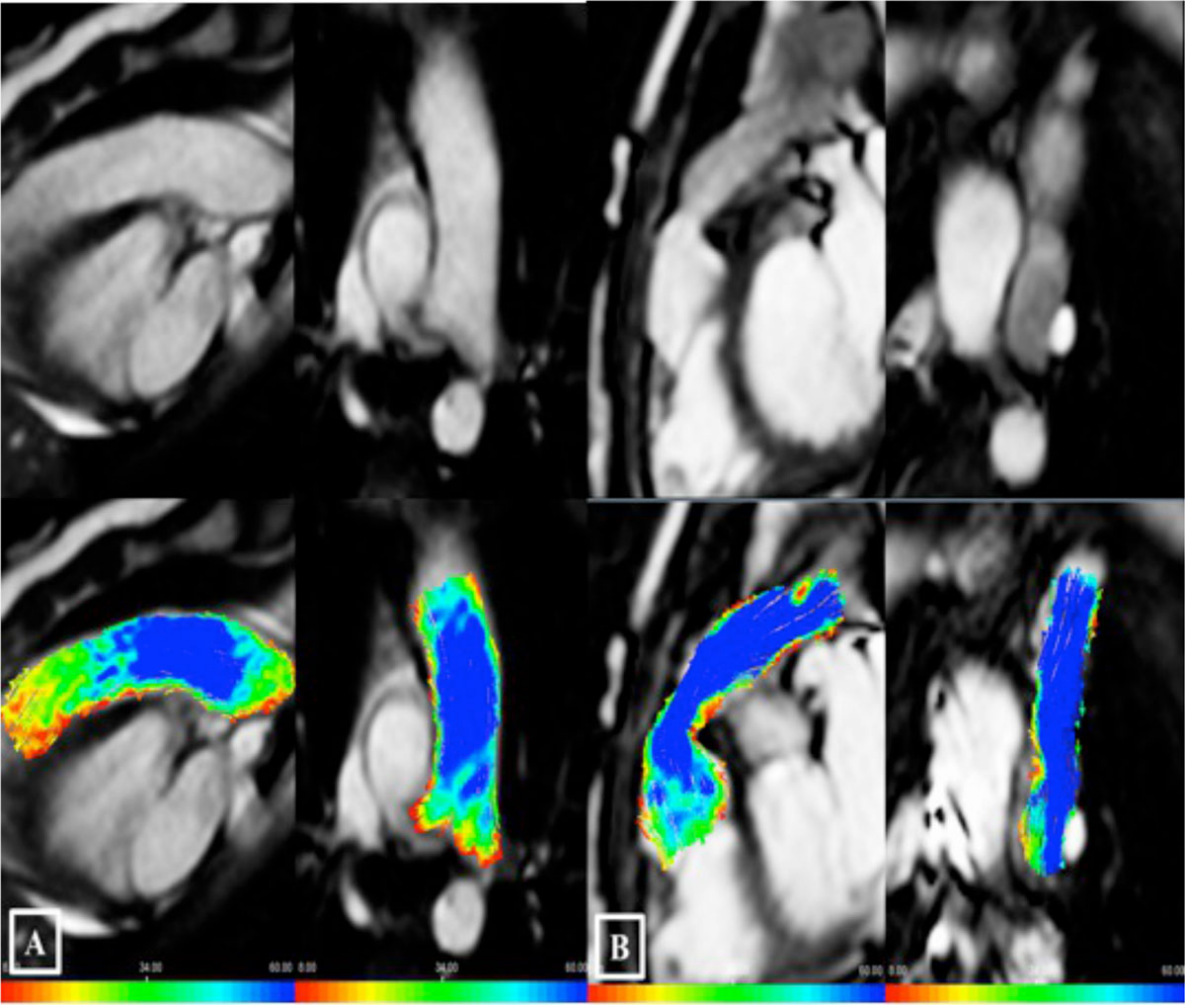
**(A) Pulmonary artery in a normal subject; upper left (sagittal view), and upper right (coronal view)**. (B) Contegra conduit; upper left (sagittal view), upper right (coronal view). Lower rows show traces of color-coded streamlines demonstrating laminar flow, no visible vortex, and no deviation of the flow from the mid line.

Patients with percutaneous implantation showed an eccentric pulmonary flow (deviation angle from the midline 31 ± 10 degree) with vortex formation (vortex size 73 ± 18%), and a significant asymmetric elevated WSR at focal regions of the conduit. (Figure 1) Vortex size was positively correlated with the pressure gradient across the valve and with the flow deviation angle. In contrast, those after surgical implantation showed a laminar pulmonary flow with no visible vortex and had symmetric, although elevated WSR in the conduit regions.(Figure 2)

## Conclusions

Pulmonary flow patterns differ significantly between patients with a surgical and percutaneously implanted bovine valve in the RVOT. Longer follow up studies will be required to determine the implications of such knowledge for prognosis and therapy.

## Funding

No disclosure.

